# Association of testosterone level with melasma in men: a case-control study in Indonesia

**DOI:** 10.11604/pamj.2022.43.194.37435

**Published:** 2022-12-16

**Authors:** Ratih Purnamasari Nukana, Luh Made Mas Rusyati, Gusti Ayu Agung Praharsini, Ketut Kwartantaya Winaya, Nyoman Suryawati, Ni Luh Putu Ratih Vibriyanti Karna

**Affiliations:** 1 Department of Dermatology and Venereology, Faculty of Medicine Udayana University, Ngoerah General Hospital, Denpasar, Bali, Indonesia

**Keywords:** Melasma, men, testosterone, low

## Abstract

**Introduction:**

melasma is a common acquired hypermelanosis which occurs mostly in face that caused by many factors. One of the pathogenesis of melasma in men is affected by testosterone. This study aimed to investigate the relationship between testosterone levels and melasma in men and its association with the severity of melasma as measured by the melasma area and severity index (MASI) score.

**Methods:**

a case-control study involving 30 subjects with melasma and 30 subjects without melasma who were treated at the outpatient clinic of Dermatovenereology of Ngoerah General Hospital from June to August 2022. Descriptive statistical analysis is to determine frequencies and percentages. Bivariate analysis was used to find any risk factor between testosterone level and melasma. Data obtained from the two groups then analyzed for correlation between the MASI score and testosterone levels.

**Results:**

mean age of the subjects in the melasma group was 43.83±6.30 and in control group was 43.80±6.09. Mean testosterone level in the melasma group (7.55±1.77) was significantly lower than the control group (21.07±6.65; p = 0.001). Subject with testosterone level ≥8.92 nmol/L has 6.9 times risk of melasma compare to control (aOR: 6.986, 95% CI 1.905-25.622; p = 0.003).

**Conclusion:**

low testosterone levels possibly have a role in pathogenesis of melasma in men.

## Introduction

Melasma is a hyperpigmentation disorder on the area that is exposed to the sun. Melasma affect all races and genders. Although more commonly reported in women, melasma also occurs in men, especially in dark-skinned individuals living in areas with high ultraviolet (UV) radiation. For male patients, melasma is a cosmetic problem with severity ranging from mild pigmentation to severe hypermelanosis and can have a major impact on quality of life. Melasma mostly affects the face, thereby affecting the appearance and causing an emotional burden and low self-confidence in their social life [1]. Epidemiological studies related to melasma in men are rarely reported because few men seek medical attention. There was only a single man who visited the Outpatient clinic of Dermatovenereology of Ngoerah Hospital for melasma in 2014 [2]. Meanwhile, Parsam *et al*. demonstrated a ratio of 3.54 : 1 for melasma in women over men [3]. The pathophysiology of melasma is multifactorial, and hormonal factor can be contributed. In women, estrogen and progesterone play an important role in the occurrence of melasma [4]. While in men, a hormonal imbalance, specifically low testosterone level can affect the occurrence of melasma [4,5]. Testosterone is a male sex steroid hormone that is mainly produced after the testes mature and is the most important androgen hormone with up to 95% produced by Leydig cells [6]. A study conducted in India show that male with melasma had lower testosterone level than normal men [4]. Meanwhile, another study conducted by Handa *et al*. show different results which is showed no statistical difference in testosterone level between men with melasma and those without melisma [5]. The aim of this study to determine the role of testosterone in melasma and relationship between testosterone and severity of melasma as measured by the MASI score.

## Methods

**Study design and setting:** this study was an analytic observational study with a case-control design. The subjects were consecutively obtained from patients visiting the Department of Dermatovenereology of Ngoerah General Hospital from June to August 2022. Ngoerah General Hospital is a tertiary referral hospital in Bali.

**Study population:** the study population are men with and without melasma. The inclusion criteria were men aged 25-55 years, Fitzpatrick skin type III - V, and exposed to the sun ≤ 6 hours dan > 6 hours. The exclusion criteria were men who underwent chemotherapy, and men who took medication caused hyperpigmentation such as phenothiazines, chlorpromazine, tricyclic antidepressants, and phenytoin within 4 weeks before the study enrollment.

**Data collection:** samples that meet the inclusion criteria, will give a questionnaire with entries that include basic data on patient characteristic: name, age, ethnic, occupation, length of sun exposure, previous medical history, medical history, family history of illness, and social history. After that, a physical examination was carried out which included vital signs, general status, and dermatological status. The diagnosis of melasma is made by history taking, clinical examination and supporting examination using wood's lamp. Determining the severity of melasma was calculated by the MASI score which was examined by the same two expert doctors throughout the study to minimize bias. Venous blood sampling taken on 08.00-11.00 Am. The blood that has been taken will be sent to the Integrated Biomedical Laboratory, Faculty of Medicine, Udayana University.

**Laboratory analysis:** the venous blood that has been taken is put into a blood tube without Ethylenediaminetetraacetic acid (EDTA). Three (3 ml) of blood was inverted 10 times until homogeneous, allowed to stand for 30 minutes until the blood was frozen, and then the blood sample was centrifuged at 3000 rpm for 10 to 15 minutes. If all serum has been collected, the serum testosterone level is measured using ELISA method.

**Definition:** variables in this study consist of three. Independent variable is testosterone level, dependent variable are melasma and MASI score. Control variable are age, Fitzpatrick skin type, length of sun exposure, smoking, alcohol consumption, diabetes mellitus, obesity, Klinefelter syndrome, Kallmann syndrome, Prader-Willi syndrome, Riehl's melanosis, post-inflammatory hyperpigmentation, history of use of sunscreens and creams containing Kligman Formula.

**Statistical analysis:** after the above data is collected data processing and analysis is carried out. The statistical analysis was conducted with the Statistical Package for The Social Sciences (SPSS) version 16. Case and control group were matched by age and Fitzpatrick skin type. Descriptive analysis using to explain demographic and clinical picture of subjects. Normality of data were tested using the Shapiro-Wilk. Relationship between the independent (testosterone) variable and the dependent variable (melasma and MASI score) used Mann-Whitney test. The correlation between variables using Spearman's rho. Determined categorical variables will be continued to bivariate analysis with chi square and multivariable analysis with logistic regression.

**Ethical considerations:** samples met the inclusion criteria, written informed consent and sign a consent form before participating in the study. This study received ethics approval by the Ethics Committee of the Faculty of Medicine, Udayana University/ Ngoerah General Hospital with approval number 839/UN14.2.2.VII.14/LT/2022.

## Results

**Demographic and clinical characteristic:** this study involved 30 men with melasma and 30 men without melasma who were matched according to Fitzpatrick's age and skin type. The mean age of the subjects in the melasma group was 43.83±6.30 and 43.80±6.09 in the control group. Family history with melasma is higher in melasma group [86.7% (n=26)] than control group [40.0% (n=12)]. Based on length of sun exposure in melasma group found that 17 (56.7%) sample exposed for > 6 hours, but in control group no one was exposed for > 6 hours. The mean duration of melasma was 6.33±2.17 years. The mean testosterone level in the melasma group was 7.55±1.77 nmol/L, while the control group was 21.07±6.65 nmol/L. The mean of MASI score was 17.85±3.42. The characteristics of the sample are described descriptively in Table 1.

**Table 1:** demographic and clinical feature of subjects in both groups

Characteristic	Control (n,%) (n=30)	Melasma (n,%) (n=30)
**Ethnicity**		
Bali	18 (60.0)	16 (53.3)
Others	12 (40.0)	14 (46.7)
**Age** (years; mean±SD)	43.80±6.09	43.83±6.30
**Occupation**		
Construction workers	0 (0.0)	13 (43.3)
Cleaning Service	7 (23.3)	0 (0.0)
Teacher	1 (3.3)	0 (0.0)
Contractor	1 (3.3)	0 (0.0)
Mechanic	2 (6.7)	0 (0.0)
Shopkeeper	2 (6.7)	0 (0.0)
Fishmonger	1 (3.3)	0 (0.0)
Security	7 (23.3)	0 (0.0)
Technician	5 (16.7)	0 (0.0)
Parking attendans	0 (0.0)	8 (26.6)
Gardener	0 (0.0)	9 (30.0)
Enterpreneur	1 (3.3)	0 (0.0)
Jobless	3 (10.0)	0 (0.0)
**Body Mass Index**		
Obesity, overweight	2 (6.7)	6 (20.0)
Normal	28 (93.3)	24 (80.0)
**Smoking**		
Yes	16 (53.3)	15 (50.0)
No	14 (46.7)	15 (50.0)
**Alcohol**		
Yes	4(13.3)	0
No	26 (86.7)	30 (100.0)
**Fitzpatrick skin type**		
IV	29 (96.7)	29 (96.7)
V	1 (3.3)	1 (3.3)
**Sun exposure (hours)**		
≤ 6	30 (100)	13 (43.3)
> 6	0 (0)	17 (56.7)
**Family history with melasma**	12 (40.0)	26 (86.7)
Yes		
No	18 (60.0)	4 (13.3)
**Distribution of lesions** Malar	-	26 (86.7)
Centrofacial		4 (13.3)
Mandibular		0 (0.0)
**Melasma Type**		
Epidermal	-	19 (63.3)
Dermal	-	4 (13.3)
Mixed	-	7 (23.3)
**Duration melasma** (years)	-	6.33±2.17
**Testosterone levelsb** (nmol/L; mean±SD)	21.07±6.65	7.55±1.77
**Masi** (mean±SD)	-	17.85±3.42

**Bivariate analysis:** in family history of melasma, we found statistically significant between two groups (p=0.006; 95% CI 1.82-23.21) with an Or of 6.5. Based on length of sun exposure, a significant difference was found between the two groups (p=0.001; 95% CI 0.19-0.47) with an OR of 0.3. Testosterone levels were also found to be significantly different in the two groups (p=0.001; 95% CI 2.71-10.29) with an OR of 5.28 (Table 2).

**Table 2:** bivariate analysis

Variable		Control (n=30)	Melasma (n=30)	Or	95%		p-value
		n (%)	n( %)		Upper limit	Lower limit	
**Age (years)**	< 43.81	14 (46.7)	15 (50)	0.87	0.31	2.41	0.79
	> 43.81	16 (53.3)	15 (50)				
**Body mass index**	Obesity, overweight	2 (6.7)	6 (20.0)	3.50	0.64	18.98	0.254
	Normal	28 (93.3)	24 (80.0)				
**Family history with melasma**	Yes	12 (40.0)	26 (86.7)	6.50	1.82	23.21	0.006*
	No	18 (60.0)	4 (13.3)				
**Smoking**	Yes	16 (53.3)	15 (50.0)	0.87	0.32	2.41	0.796
	No	14 (46.7)	15 (50.0)				
**Alcohol**	Yes	4 (13.3)	0 (0)	1.97	0.51	7.63	0.505
	No	26 (86.7)	30 (100)				
**Sun exposure (hours)**	≤ 6	30 (100)	13 (43.3)	0.3	0.19	0.47	0.001*
	> 6	0 (0)	17 (56.7)				
**Fitzpatrick skin type**	IV	29 (96.7)	29 (96.7)	1.00	0.60	16.76	1.00
	V	1 (3.3)	1 (3.3)				
**Testosterone (nmol/L)**	≤ 8.92	0 (0)	23 (76.7)	5.28	2.71	10.29	0.001*
	> 8.92	30 (100)	7 (23.3)				

* P value is significant if <0.05

**Multivariable analysis:** the variables that had significant results in the bivariate analysis were family history of melasma, duration of sun exposure and testosterone level. These variables will be analyzed by multivariable analysis to determine which variables had the most influence. Based on our study, it shows that testosterone levels are the most significant risk factor for melasma in men. Testosterone level ≤ 8.92 nmol/L has 6.9 times risk of melasma in male patients compare to those with > 8.92 nmol/L (aOR: 6.986, 95% CI 1.905-25.622; p = 0.003) (Table 3).

**Table 3:** multivariable analysis

Independent variable	P value	Adjusted odd ratio (AOR)	95% CI
**Family history melasma**			
Yes	0.023	5.731	1.273-25.797
No		1	
**Sun exposure**			
≤ 6 hours	0.070	1	0.867-37.040
> 6 hours		5.667	
**Testosterone level**			
≤ 8.92 nmol/L	0.003	6.986	1.905-25.622
> 8.92 nmol/L		1	

* P value is significant if <0.05

**Correlation of melasma and Masi score:** the spearman correlation was conducted between to analyze the relationship Masi score and testosterone levels. There was a statistically significant correlation between the MASI score and testosterone levels with a strong negative correlation coefficient (r=-0.933; p=0.001).

## Discussion

The etiology of melasma is still unknown, but melasma can aggravate by many factors. One of them is caused by hormonal influences. In men, unbalanced testosterone levels can affect the occurrence of melasma. In our study, the mean testosterone level in melasma group was found to be significantly lower (7.55±1.77 nmol/L) than control group (21.07±6.65 nmol/L) (p=0.001). This is comparable with the study conducted by Sialy *et al*. which found a lower mean testosterone level in melasma (11.97 ± 0.8^4^nmol/L) than normal male (20.51 ± 2.25 nmol/L) with a statistically significant difference (p < 0.002) [4]. This is also accordance with the studies involving men with melasma by Sarkar *et al*. in India and Ogita *et al*. in Japan who found lower testosterone levels in the melasma group than the control group. This highlights the role of testosterone in the pathogenesis of melasma [7,8]. However, in contrast to these studies, Handa *et al*. found that there is no relationship between hormonal factor and melasma. They found that testosterone levels in the melasma group were higher (15.58±4.97 nmol/L) when compared to the control group (13.09±5.48 nmol/L), but the difference was not significant (p=0.07). This is may be due to a small number of samples, and 60% of the subjects in the melasma group used mustard oil on the face. Mustard oil is known to have a photosensitizer effect. This material is the most common risk factors for melasma in the study [5]. Our study demonstrated that a low testosterone levels of < 8.92 nmol/L had a 5.28 times higher risk of developing melasma compared to men who had higher testosterone levels. Melasma is more common in dark-skinned individuals, such as Asian and African-American race who live in areas with intense ultraviolet radiation exposure. In this study, there were 60 subjects from four different ethnic groups in Indonesia, specifically the Balinese, Javanese, West Nusa Tenggara, and East Nusa Tenggara. Balinese ethnicity were predominant in both groups, but no significant difference was found (p=0.694), its may be due to the same root of race, namely Asian race. Wu *et al*. showed that melasma is more common in Asian [9]. It also correlates to skin color according to Fitzpatrick skin type. Melasma is common in individuals with Fitzpatrick skin types IV- VI. In melasma group were found 96.7% (n=20) had Fitzpatrick skin type IV. This is comparable to a study conducted by Handa *et al*., who demonstrated that Fitzpatrick skin types IV more commonly experienced melasma (66%) when compared to Fitzpatrick skin types V (14%), Fitzpatrick III (18%) and Fitzpatrick II (2%) [5]. The majority of subjects in this study were in the age range of ≥41 years (mean 43.81±6.14 years). This study is accordance with study conducted by Sialy *et al*. who demonstrated male melasma patients in the range of 20 to 40 years [4]. This study is also in accordance with a study Pichardo *et al*. who conducted an epidemiological study of melasma in men working as farmers. In their study, the highest age range was > 31 years (51.3%) [10]. These studies highlighted that melasma may begin to appear in the third or fourth decades of life [11].

The three dominant distribution patterns of melasma on the face are centrofacial, malar, and mandibular. In this study, the malar pattern was the most common pattern found in subjects with melasma with 86.7% (n=26), followed by centrofacial patterns in 13.3% (n=4). This finding is similar with the results of the study conducted by Sarkar *et al*. which found 61% malar pattern, 29.3% centrofacial and 9.7% mandibular melasma pattern in men [12]. Accordance with the study conducted by Amrutha *et al*., 63% of subjects had malar pattern melasma, 30% had centrofacial melasma and the rest had mandibular pattern [13]. Wood's lamp examination in this study demonstrated epidermal type in 63% of cases, while mixed type and dermal type were found to be 23.3% and 13.3%, respectively. This is in accordance with two studies conducted by Sarkar *et al*. which found that the most common melasma was epidermal type in 68.3% (n=28) followed by mixed type in 22.0% (n=9) and dermal type in 4 subjects 9.7% (n=4). This study was also supported by Handa *et al*. who demonstrated the predominance of epidermal melasma followed by mixed type and then dermal type [5,12]. Based on length of sun exposure, there was a significant difference between the two groups (p=0.001). The duration of sun exposure in the control group was ≤ 6 hours in 100% (n=30), while in the melasma group, the duration of sun exposure was ≤ 6 hours in 4.3% (n=13), > 6 hours in 17 subjects 56.7% (n=17). This finding is accordance with the study conducted by Parsam *et al*. highlighting that the longer exposure to the sun, the more severe the degree of melasma. In his study, subjects exposed for > 6 hours had a mean MASI score of 8.57±3.83 while those exposed for < 6 hours had lower MASI score of 5.4±4.24 with a statistically significant difference (p=0.0006) [3].

Ultraviolet light has been known to play a role in increasing the expression of the deoxyribonucleic acid (DNA) methyltransferase enzyme, thereby increasing the DNA methylation process. This will lead to an increase in pro-inflammatory and pro-melanogenic genes that play a role in the pathogenesis of melasma [9,14]. In addition, ultraviolet radiation, especially ultraviolet B (UVB) promotes keratinocytes to induce melanocyte proliferation and melanogenesis by secreting stem cell factor (SCF), basic fibroblast growth factor (bFGF), interleukin-1, endothelin-1, inducible nitric oxide synthetase, melanocyte-stimulating hormone, adrenocorticotropic hormone, and prostaglandin E2. Sun damage can also induce the release of melanogenic cytokines, including SCF and hepatocyte growth factor from dermal fibroblasts, thereby affecting the development of hyperpigmentation in the overlying epidermal tissue [15]. Another risk factor that plays a role in the development of melasma is genetics. Tamega *et al*. reported that 56.3% of 302 patients with melasma had a family history of melasma [16]. Similarly, Handel *et al*. in their study involving 207 patients with melasma showed that 61% had a family history of melasma [11]. Our study is also in concordance with the two previous studies. In this study, the majority of subjects (86.7%) in the melasma group had a family history of melasma in comparison to the control group with only 40% (p=0.001). The testosterone is involved in the pathogenesis of melasma. At normal levels, this hormone can reduce the level of cyclical activity of adenosine monophosphate and tyrosinase in cells so that it plays a role in preventing melasma. In the presence of low levels of testosterone, the activity of adenosine monophosphate and tyrosinase increases, causing melasma [6]. A study conducted by Bischitz *et al*. stated that testosterone can cause melasma because it causes elongation, enlargement and increase in the number of dendritic cells and melanocytes. This will cause an increase in the number of free melanin cells and induce melasma [17]. The MASI score is a validated score used to measure the degree of facial hyperpigmentation. This instrument is a numerical score that is calculated as the cumulative score of the areas of pigmentation and homogeneity located on the forehead, chin, right and left malar cheeks [18]. Based on the data obtained from subjects with melasma, the mean MASI score was 17.85 with a standard deviation of 3.42, indicating that the subjects mostly had mild and moderate melasma. The correlation analysis of testosterone levels with the MASI score in this study showed a strong and statistically significant negative correlation (r=-0.933; p=0.001). To the best of our knowledge, this is the first study linking testosterone levels with MASI scores. The strength of this study is that, it is the first study that correlates between testosterone level and MASI score and investigate relationship between testosterone level and melasma in Indonesian men. The limitations of this study are a single drawing of blood for sampling and a single center involved. Further studies are needed with serial testosterone levels by measuring testosterone before and after melasma therapy to better ascertain the effect of testosterone on the occurrence of melasma.

## Conclusion

This study determined the testosterone levels of men with melasma are lower than men without melasma. There is a negative correlation between testosterone levels and MASI scores in men with melasma.

### What is known about this topic

- The etiology of melasma is multifactorial and melasma in male is underreported;
- Hormones are one of the pathophysiology’s of melasma.

### What this study adds

- Low serum testosterone level might be a risk factor for melasma in men;
- A negative correlation between serum testosterone levels and MASI scores in men with melasma.
